# Ethnomedicinal Knowledge of Plants Used in Nonconventional Medicine in the Management of Diabetes Mellitus in Kinshasa (Democratic Republic of the Congo)

**DOI:** 10.1155/2023/4621883

**Published:** 2023-09-20

**Authors:** Bashige Chiribagula Valentin, Okusa Ndjolo Philippe, Manya Mboni Henry, Félicien Mushagalusa Kasali

**Affiliations:** ^1^Department of Pharmacy, Laboratory of Therapeutic Chemistry and Analysis of Natural Substances, Faculty of Pharmaceutical Sciences (Université de Lubumbashi), 27 Avenue Kato, Commune Kampemba, Lubumbashi, Congo; ^2^Department of Pharmacy, Faculty of Pharmaceutical Sciences and Public Health, Université Officielle de Bukavu (UOB), P.O. Box: 570, Bukavu, Commune of Kadutu, Av. Karhale, Congo

## Abstract

**Background:**

People with diabetes, herbalists, and traditional medicine practitioners (TMPs) from Kinshasa use plants to treat diabetes, but no study has inventoried the plants used by these populations. The present study was conducted to identify the plants used in Kinshasa to treat diabetes mellitus.

**Methods:**

The survey conducted in the form of a semistructured interview between March 2005 and August 2006 made it possible to collect ethnobotanical information from people with diabetes (*n* = 126), herbalists (*n* = 80), and TMPs (*n* = 120).

**Results:**

The 326 subjects consulted (sex ratio *M*/*F* = 0.6, age 51 ± 7 years, and experience: 17 ± 5 years) provided information on 71 plants, most of which are trees (35%), belonging to 38 families dominated by Fabaceae (19.7%) and indicated in 51 other cases of consultation dominated by malaria (12%). From these 71 plants derived, 86 antidiabetic recipes were administered orally, where the leaf is the most used part (>50%) and the decoction (>46%) is the most common mode of preparation. This study reports for the first time the antidiabetic use of 11 species, among which *Tephrosia vogelii*^X^ (0.08), *Chromolaena corymbosa*^X^ (0.06), and *Baphia capparidifolia*^X^ (0.06) present the highest consensus indexes (CI) and *Marsdenia latifolia*^W^ (UV*p* = 0.08) and *Rauvolfia mannii*^X^ (UV*p* = 0.06) present the highest UVs.

**Conclusion:**

The results show that Kinshasa people treat diabetes using several plants. Some are specific to the ecological environment; others are used in other regions. Pharmacological studies are underway to assess the therapeutic efficacy of these plants.

## 1. Introduction

Diabetes mellitus (DM) is a metabolic and chronic disease involving inappropriately elevated blood glucose levels (hyperglycemia). Hyperglycemia alone can impair pancreatic beta-cell function and contributes to impaired insulin secretion. The body cannot produce and secrete sufficient insulin hormone or use it effectively. This insulin deficiency leads to elevated blood glucose levels and decreases carbohydrate and protein metabolism [1, 2].

Worldwide, 537 million adults (20–79 years old) live with diabetes. This number is expected to rise to 643 million by 2030 and 783 million by 2045. More than 3 out of 4 adults with diabetes live in low-income countries like DR Congo, and diabetes has been responsible for 6.7 million deaths in 2021, i.e., one end every 5 seconds [1]. Cardiac, vascular, neurological, and renal damage and neuropathy may occur without appropriate treatment. Treatment includes diet, exercise, and medication [3]. Given that most cases occur in low-income countries, where a significant fraction of the population resorts to traditional medicine, which essentially uses plant resources, medicinal plants constitute a credible alternative in the fight against diabetes mellitus.

Natural products have been shown to play an essential role in regulating pathophysiological signaling pathways, particularly in diabetes [4]. Over 800 plant species showing hypoglycemic activities can be vital sources in discovering and developing new types of antidiabetic molecules [5].

Medicinal plants have a high potential to treat various ailments due to the presence of their significant bioactive phytoconstituents. Certain plants are rich sources of compounds reputed to be antidiabetic such as flavonoids, alkaloids, phenolic compounds, and tannins, improving pancreatic tissues' efficiency by increasing insulin secretion or decreasing intestinal glucose absorption [6].

The World Health Organization insists that scientists conduct ethnomedicinal, ethnobotanical, and ethnopharmacological investigations to record and preserve traditional knowledge, create databases, and validate scientifically traditional claims from the perspective of developing improved medication [7].

In the Democratic Republic of the Congo, a country with similarities to other developing countries, data on the prevalence of diabetes mellitus and the use rate of medicinal plants in their therapeutic load is not sufficiently available. Although less systematic, some studies have reported presumed antidiabetic plants in Kinshasa [8, 9].

This study completes the data reported in these studies while highlighting plants used by TMPs, diabetics, and herbalists in Kinshasa.

## 2. Methods

### 2.1. Experimental Framework

The city-province of Kinshasa is located between 4°18′ and 4°25′ south latitude and between 15°18′ and 15°22′ east longitude. It is bounded to the north and east by the province of Kwilu, to the south by the province of Congo Central, and to the west by the Republic of the Congo, with an average altitude of 300 m above the sea. Kinshasa city's climate is tropical. It is characterized by a long rainy season lasting for 8 months (October–July), discontinuous between January and February, followed by August and September in favor of the short dry season. The vegetation of Kinshasa consists of degraded primitive forests, savannahs, and aquatic and semiaquatic formations in the valleys and Pool Malebo. It belongs to the Guinean-Congolese region, the Congolese basin domain, and the Congolese-Zambezian transition sector [10].

### 2.2. Ethnobotanical Data Collection

This cross-sectional descriptive ethnobotanical study was conducted between March 2005 and August 2006 through semistructured interviews based on a questionnaire. The discussion focused on knowledge about plants used to manage diabetes mellitus. Three groups of subjects were consulted: people with diabetes, herbalists, and TMPs from Kinshasa.

People with diabetes were met in 4 health zones of the city of Kinshasa: Bumbu, Kalamu, Limete, and Makala, covering the biomedical centers for the care of people with diabetes in Kinshasa supervised by BDOM (diocesan office of biomedical works). The reference health centers (RHCs) Libundi, Bondeko, Saint Clément, 2e Rue, the Bondeko clinic, and the Saint Joseph General Reference Hospital were concerned.

The herbalists consulted during this study were met in 5 popular markets in Kinshasa, the central market, the Selembao market, the Matadi Kibala market, the Mariano market, and the UPN market.

The TMPs were met in 4 communes of Kinshasa (Bumbu, Kalamu, Limete, and Makala). These municipalities are covered by the health zones selected during the survey of people with diabetes. TMPs were reached by snowballing from the TMPs provided by the population in each area concerned, and the investigations were carried out in Lingala. The listed plant species were collected and placed in herbariums, then compared to the reference herbariums from Kisantu (National Agricultural Study and Research Institute), to identify the scientific names. These were then formatted according to the Plants of the World online database: Plants of the World Online/Kew Science (https://powo.science.kew.org/) or African Plant Database (https://africanplantdatabase.ch/) or the World Flora Online (https://www.worldfloraonline.org/).

### 2.3. Data Analysis

Three ethnobotanical indexes were calculated to assess the significant species: the usual value (UV), the fidelity index (FI), and the consensus index (CI). The following formula determined the usual value (VU): VU = (∑Ui)/Ni, where Ui is the number of uses mentioned by an informant for a plant (organ) and Ni = the number of informants who cited the plant. The following formula calculated the consensus index on the plant (organ) (CI): CI= Np/*N*, where Np is the number of people who cited the plant (organ) and N is the number of people who were consulted in the study. The fidelity index of the recipe was calculated by the formula FI = nr/Np, where nr = the number of people who cited the recipe and Np is the number of people who cited the plant. Apart from the 3 ethnobotanical indexes mentioned above, the relative citation frequency (RCF) was also determined. It was calculated by the formula *F*_CR_ = nx100/*N* with *n* being the number of occurrences of the factor examined and *N* = total number of the population concerned. This factor was used to quantify various factors analyzed in this study, except those involved by the ethnobotanical indexes.

In this study, UV makes it possible to evaluate the medicinal importance of a plant in the environment; CI makes it possible to identify the level of consensus of the population on the use of a species in the management of diabetes mellitus; and FI makes it possible to establish the level of loyalty that emerges among informants on an antidiabetic recipe.

## 3. Results

### 3.1. Ethnobotanical Profile of Inventoried Plants

**Figure 1:**
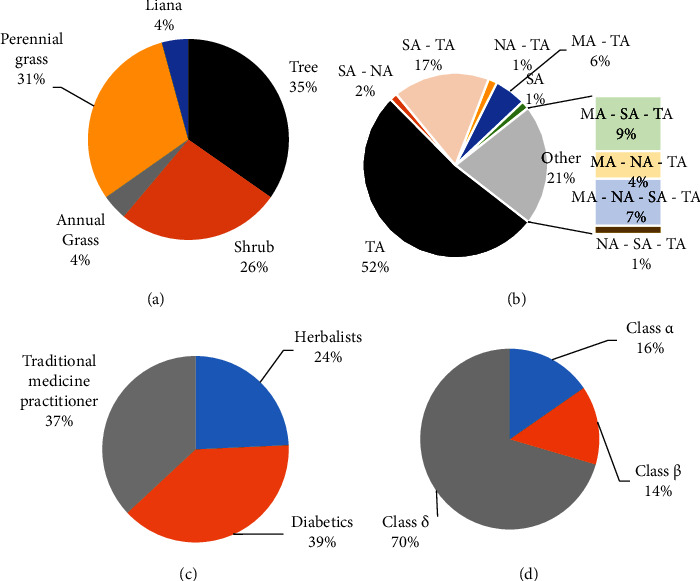
Morphological (a) and geographical (b) type of plants, category of informants (c), and situation of the plant with the literature (d). NA: North Africa; CA: Central Africa; TA: tropical Africa; SA: Southern Africa; MA: Madagascar; plants with pharmacological study concerning diabetes: class *α*; plants without ethnobotanical or pharmacological study related to diabetes: class *β*; plants for ethnobotanical use but without pharmacological research concerning diabetes: class *δ*.

Seventy-one plants were inventoried during this study. These plants were informed by 3 types of informants, herbalists (class Y), diabetics (class X), and TMPs (class W). The coexistence of these 3 types of informants gave rise to two other classes, the class of plants common to the 3 sources (class Z) and plants familiar to herbalists and TMPs (class V). As such, the results show that 16 plants are from diabetics, 7 plants are from herbalists, 22 are from TMPs, 6 are from both herbalists and TMP, and 20 are from TMPs, herbalists, and diabetics (Table 1). It should be noted, however, that the TMP class provided the largest most significant number of species, i.e., 31% (Figures 1(c) and 1(d)).

**Figure 2:**
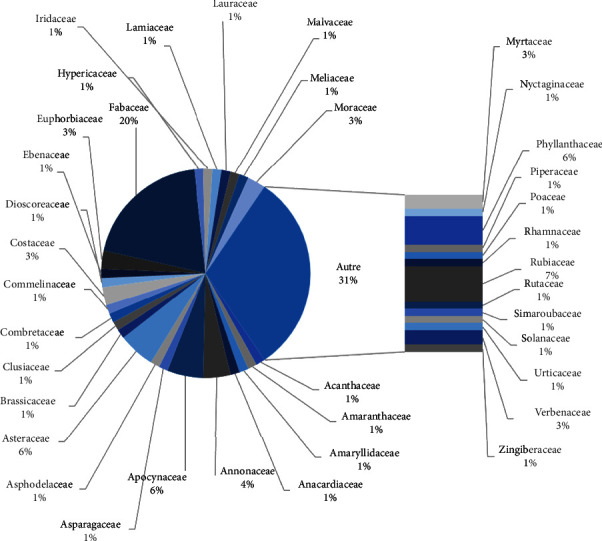
Botanical family of listed plant species. Wp: whole plants, Ro: roots, Tb: tubers, and Nr: not reported.

**Figure 3:**
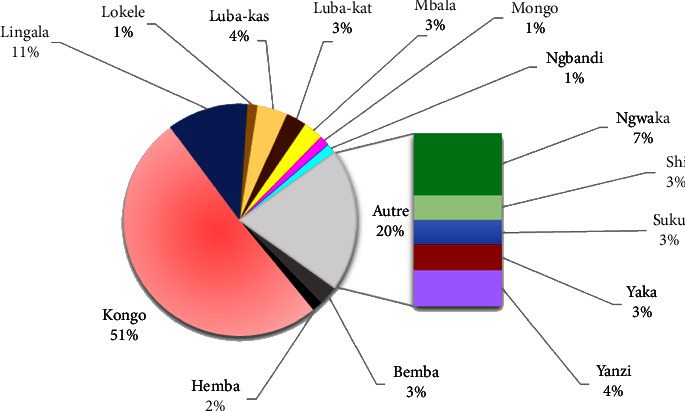
Languages of names of inventoried plants.

Each of these plants is named in one of the 15 Congolese ethnic groups reported in this study, of which Kikongo (50.7%) and Lingala (11.3%) occupy the first two places (Figure 2). These plants belong to 38 families dominated by the Fabaceae (19.7%), followed, respectively, by Rubiaceae Juss (7.0%), Apocynaceae Juss (5.6%), Asteraceae Bercht and J. Presl (5.6%), and Phyllanthaceae Martinov with 5.6% (Figure 3). These plant taxa are mostly trees (35%) or perennial herbs (31%) endemic to tropical Africa (Figures 1(a) and 1(b)). Among the 71 inventoried species, only *Persea americana* Mill (30.67%), *Senna alata* (L.) Roxb (30.67%), *Garcinia kola* Heckel (18.40%), *Piliostigma reticulatum* (D.C.) Hochst (17.79%), and *Vachellia karroo* (Hayne) Banfi and Galasso (17.79%) showed a relative citation frequency >15% (Table 1).

85% of identified medicinal plants in the present study have been previously used in local medicines as antidiabetic plants (13%). However, 72% have demonstrated an antihyperglycemic effect. Nevertheless, this study highlights 11 plant species never reported before as antidiabetic plants, among which *Tephrosia vogelii*^X^ (*F*_CR_ = 7.98), *Chromolaena corymbosa*^X^ (*F*_CR_ = 6.44), and *Baphia capparidifolia*^X^ (*F*_CR_ = 5.52) are the most cited (Table 1).

### 3.2. Ethnomedical Profile of Inventoried Plants

**Figure 4:**
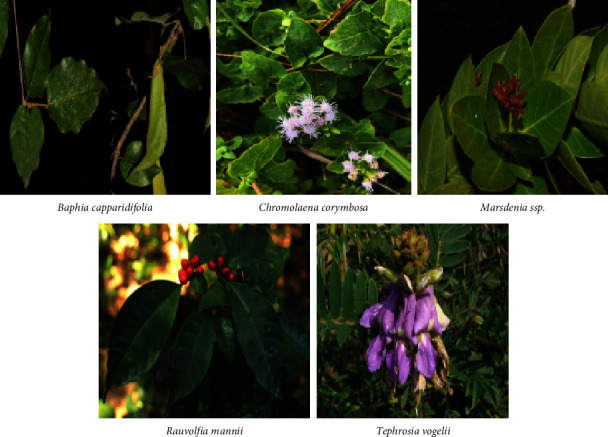
Important plant species identified as antidiabetic in Kinshasa (figures collected from https://www.inaturalist.org).

The 71 plants inventoried during this study are used in 86 antidiabetic recipes, of which 80 recipes use one plant, and 6 combine two plants (Table 2). The administration of the two types of recipes is essentially done orally, the leaf is the most used organ (50 and 58.3%), and decoction is the most predominant mode of preparation with 46.3 and 66.7% (Figure 4).

Figure 4 indicates that *Baphia capparidifolia*, *Chromolaena corymbosa*, *Marsdenia latifolia*, *Rauvolfia mannii*, and *Tephrosia vogelii* are among the critical plant species, according to their consensus index and use value.

In these recipes (R81–R86), we can observe the associations of leaves-leaves, roots-roots, and leaves-roots, representing half of these associations. Only 12 of the 71 plants are involved in the recipes combining two plants. For these recipes of plants in association, the treatment duration varies between 30 and 45 days, and it is the combination of *Albizia adianthifolia* (leaves) and *Annona senegalensis* (roots) with an RCF of 4.6 which is the most cited: R85 (Table 3).

The consensus indexes of the identified antidiabetic recipes (ICR) varied between 0.01 and 0.31, with the highest value observed for the recipes R61, based on *Persea americana*^X^ leaves, and R72, based on leaves of *Senna alata*^X^.

For the 11 plants reported for the first time by this study as antidiabetics, the ICRs vary between 0.08 and 0.01, with *Tephrosia vogelii*^X^ (0.08), *Chromolaena corymbosa*^X^ (0.06), and *Baphia capparidifolia*^X^ (0.06) showing the highest values. Usual plant UVp values vary between 0.02 and 0.12; the highest values were observed in *Annona senegalensis*^W^, *Nauclea latifolia*^Y^, and *Erythrina abyssinica*^W^ with an UVp = 0.12 each. Note also that in the group of 11 taxa reported for prayer times by this study, UVp varies between 0.02 and 0.08; these are *Marsdenia latifolia*^W^ (UVp = 0.08) and *Rauvolfia mannii*^X^ (UVp = 0.06) which presented the highest values in this class (Table 2).

**Figure 5:**
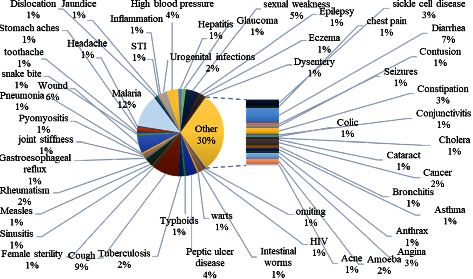
Other diseases are cured by the plants inventoried.

The 71 plants inventoried are involved in 51 other causes of consultation, including malaria (12%), cough (9%), diarrhea (7%), wounds (6%), and sexual weakness, which occupy the first 5 places. The other pathologies are below the 5% mark (Figure 5).

### 3.3. Sociodemographic Profile of Subjects Consulted

The surveyed and consulted during this study were either diabetics (38.7%) or herbalists (24.5%) or practitioners of traditional medicine (36.8%), primarily women (62.3%) whose majority age is between 48 and 58 years old. Still, the extremes are 18 and 70 years old. They mainly live either in the municipality of Kalamu (29.8%) or in Makala (29.4%), and their level of education is, on average, secondary (47.2%). They mainly exercise 4 types of profession, the most representative of which is either commerce (28.5%) or liberal work (24.2%). In most cases, they have experienced more than 11 years of use of medicinal plants in managing diabetes (Table 4).

**Figure 6:**
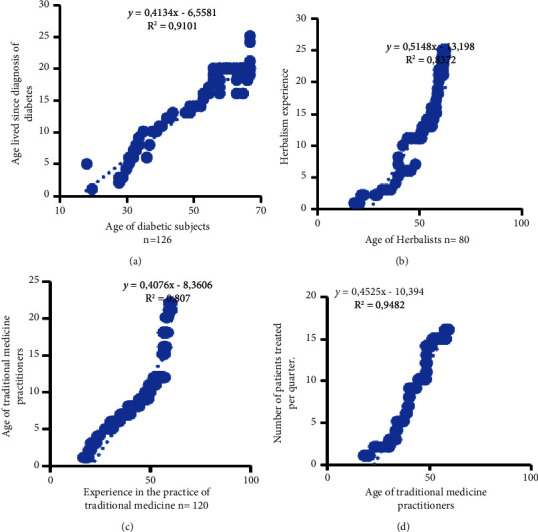
Correlations between Y-axis and X-axis variables of the characteristics of the subjects surveyed: Age lived since diagnosis of diabetes and age of diabetic subjects n=126 (a); Herbalism experience and age of herbalists n= 80 (b); Age of traditional medicine and practitioners (c); and Number of patients treated per quarter and age of traditional medicine practitioners (d).

We also sought to determine the correlation between a few variables that characterize the subjects consulted within the framework of this study. The results show a positive linear correlation (*R* = 0.95, *y* = 0.4134x-6.5581) between the age of diabetic subjects and the number of years they have lived with diabetes. Similarly, a positive linear correlation is also observed between the age of herbalists and their years of experience (*y* = 0.5148x − 13.198, *R*^2^ = 0.8372). In the same way, we noted a positive between the TMP age of the TMPs and the profession's expertise (*y* = 0.4076x − 8.3606, *R*^2^ = 0.807) and their age with the number of patients they receive per quarter, *y* = 0.4525x − 10.394, *R*^2^ = 0.9482 (Figure 6).

## 4. Discussion

The Congolese, particularly the flora of Kinshasa, have plants that traditional medicine practitioners and the general population use to manage diabetes. This study was interested in inventorying these plants and presenting their ethnobotanical and ethnomedicinal profiles. The study reported 71 taxa used in 86 recipes to treat diabetes mellitus.

These 71 plants are primarily trees from the Fabaceae family, endemic to tropical Africa, and are named mainly in Kikongo (Table 1 and Figures 2 and 3). These results are in agreement with the literature. Indeed, Fabaceae is reported to be the most prominent family of trees in Africa's tropical and dry forests [113]. This importance of Fabaceae is observed both within the plant kingdom [114] and within African medicinal plants in particular [115]. The numerical predominance of Fabaceae in sub-Saharan Africa has been attributed to their ability to scavenge atmospheric nitrogen, allowing them to grow in nutrient-poor and rich soils [116]. In our experimental framework, no accessible study has addressed the question of the preponderance of a botanical family over all the taxa used in traditional medicine in Kinshasa. Such a study would be desirable. However, the analysis of some ethnobotanical studies in Kinshasa shows that the Fabaceae constitutes one of the three most evoked families. Some previous studies in the Kinshasa reported the predominance of certain botanical families over others. As an illustration, a study on ethnopharmacological surveys of plants used in female intimate baths [116] mentioned the predominance of Rubiaceae (37%) and Fabaceae (11%). In contrast, another study on plants used to treat the symptoms of tuberculosis [117] reported the predominance of Fabaceae, Apocynaceae, and Lamiaceae, each with an FCR of 8.3%. In the same way, FCR = 47% and FCR = 12.7% were found respectively in the families of Rubiaceae and Fabaceae for a study on plants sold on the Kinshasa market [118], and an FCR of 7.7% in Fabaceae, Rubiaceae and Zingiberaceae for medicinal plants of the Lukunga district [119]. There are also studies done on the management of diabetes in the region. This is the case of a survey carried out in the Kimbanseke and Selembao communes: *n* = 21, Rubiaceae *F*_CR_ = 33.3%, Fabaceae *F*_CR_ = 19.1% [9], or the study in Kwango and Kinshasa: *n* = 68, Fabaceae *F*_CR_ = 19.1% [8]. It would nevertheless be interesting to carry out an inventory of plants known to be medicinal in the DRC to highlight their specific ethnobotanical characteristics. This concern has not been the subject of this study, which nevertheless contributes to highlighting the need for such data.

The literature review carried out on 71 taxa shows that it is possible to group the plants into 3 classes: species for which no ethnobotanical or pharmacological information is available (class *α*), those with ethnopharmacological use without any scientific evidence (class *δ*), and species for which scientific evidence is available (class *β*) (Figure 1(d)). The fact that 70% of species found are reported to be antihyperglycemic *in vivo* models (using rats) reinforces the credibility of the information obtained from our study. It suggests a high probability of finding those with antihyperglycemic activity among the 30% of the remaining species.

The 6 plant species whose antidiabetic ethnomedicinal knowledge is reported for the first time during this study and for which no pharmacological antidiabetic activity survey is registered in the accessible literature to date are *Acalypha paniculata, Baphia capparidifolia, Chromolaena corymbosa, Crotalaria medicaginea, Platymitra arborea,* and *Rauvolfia mannii*. On the other hand, 11 medicinal plants, including *Antidesma venosum, Costus phyllocephalus, Crinum ornatum, Diospyros heudelotii, Gladiolus gregarius, Lippia multiflora, Millettia drastica, Mitragyna stipulosa, Palisota schweinfurthii, Tephrosia vogelii,* and *Terminalia mollis*, have been quoted in traditional medicine as treating diabetes mellitus in various countries. However, they are not yet scientifically validated (Table 1). In both cases, these plants constitute a great richness in the database for future preclinical investigations. However, the antidiabetic evaluation of *Costus lucanusianus* has been validated after several research attempts; no study has confirmed the local antidiabetic use of this plant.

**Figure 7:**
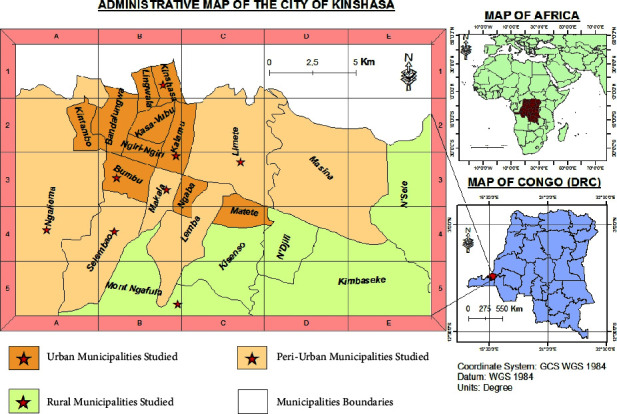
Map of Kinshasa city with different sites where data have been collected.

Previous studies on traditional healers and medicinal plant vendors have identified some species used in Kinshasa city (Figure 7) to manage diabetes mellitus. Thus, *Abelmoschus esculentus*, *Albizia adianthifolia*, *Alchornea cordifolia*, *Azadirachta indica*, *Bridelia ferruginea*, *Catharanthus roseus*, *Costus phyllocephalus*, *Cymbopogon citratus*, *Dysphania ambrosioides*, *Erythrina abyssinica*, *Ficus benghalensis*, *Gymnanthemum amygdalinum*, *Lippia multiflora*, *Morinda lucida*, *Nauclea latifolia*, *Phaseolus vulgaris*, *Phyllanthus niruri*, *Psidium guajava*, *Schwenckia americana*, *Senna alata* [8], *Annona senegalensis* [8, 73, 119], *Garcinia kola* [73, 119] *Gladiolus gregarius* [119], *Monodora myristica* [120], and *Persea americana* [8, 118] have been cited.

Concerning ethnobotanical indexes, it should be noted that for an ethnobotanical study targeted towards a specific pathology, the relative frequency of citations corresponds to the consensual citation index of the plant, which translates the consensus reached around a particular plant species on targeted use. There is a higher consensus for the 11 taxa not studied on using *Tephrosia vogelii* leaves (CIp = 0.08) as an antidiabetic than any other species of the same category (Table 2). This precedence which may result from this ethnobotanical index (CIp) is, however, to be put into perspective by the fact that a single category only told the plant of the subjects consulted, namely, people with diabetes, unlike, in particular, the leaves of the *Crinum ornatum*^Z^ species which present the weakness of ethnobotanical index whose value is twice lower than that of *Tephrosia vogelii* (CIp = 0.04), but whose strength lies in the fact that it was informed simultaneously by the three sources: diabetics, TMPs, and herbalists, which reinforces the consensus around its use as an antidiabetic in the study environment.

The study population uses three plant species most because of their highest UV of the series (0.12). These are *Annona senegalensis, Erythrina abyssinica*, and *Nauclea latifolia* (Table 2). This justifies their lower usual value of 0.02 (Table 2). All of its plants belong to the taxa category, whose ethnobotanical and pharmacological knowledge of diabetes has previously been reported. Therefore, the most common plants in the study would not be the most interesting in the context of this study, which aims to enhance the ethnopharmacological knowledge of Kinshasa. If, in most cases, each plant inventoried during this study is also known and used by our resource persons for at least one other pathology, it should be emphasized that this is not the case with *Tephrosia vogelii*^X^ and *Platymitra arborea*^Y^ for which our informants only use it in the management of diabetes mellitus.

Malaria (FCR = 12%), cough (FCR = 9%), and diarrhea (FCR = 7%) constituted the 3 first pathologies, apart from diabetes, for which the plants listed during this study are used. These pathologies are among the 10 most deadly pathologies in the DRC [121], suggesting that traditional medicine from Kinshasa will likely contribute to managing specific pathologies of concern in DR Congo. It would be interesting to carry out studies to validate this ethnopharmacological knowledge to rightly understand the assistance of conventional medicine from Kinshasa in managing pathologies such as diabetes.

The 71 inventoried plant taxa made it possible to list 86 antidiabetic recipes, 80 of which use a single plant (Table 2), and 6 recipes combine 2 simultaneous medicinal plants (Table 3). Nonetheless, considering all previous ethnopharmacological studies conducted in DR Congo [8, 15, 38, 48], this one reports antidiabetic recipes of the plants used in association for the first time.

**Figure 8:**
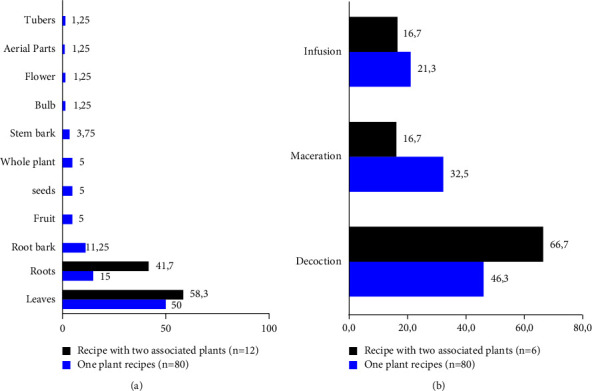
Parts used (a) and mode of preparation of antidiabetic recipes (b).

Of all the antidiabetic recipes using a single plant and reported during this study, the leaf and the decoction constitute the most used organ and mode of preparation. These results (Figure 8) are in agreement with the studies carried out on the plants of Kisangani: leaf (57.6%), decoction (78.8%), and *n* = 33 [38], and plants of DR Congo gathered in a review article, leaves 39.2%, decoction: 60.5%, and *n* = 213 [52]. Some disparities are nevertheless observed in a study in South Katanga, where the decoction (62.5%) was found to be the primary way of preparation and the root was found to be the most used organ: 41.3%, *n* = 95 [15]. The same is true in the study carried out simultaneously in Kwango and Kinshasa (*n* = 68), where the leaves at 65% proved to be the most used organ and maceration with 63% was found to be the most common mode of preparation solicited [8]. The herbal antidiabetic recipes used in Kinshasa align with the national trend, unlike those used in Lubumbashi, the country's second-largest city.

According to the subjects consulted, using a decoction would aim to extract and activate the active ingredient. This is a mixed idea, especially since this extraction procedure is more beneficial than harmful. Indeed, as much as it could facilitate the release of certain active principles often present in the plant in the form of glucosides, it could not only release specific toxic secondary metabolites such as cyanogenic glycosides [122] but also deteriorate the active ingredient when the latter is thermolabile [123–125]. Therefore, this practice remains to be assessed case-by-case, and only experimental work could determine its fair value.

About the various correlations established on specific characteristics of the subjects consulted (Figure 8), this study shows that the older the diabetic issues, the longer they have lived with diabetes, which suggests that most of the subjects consulted would be diabetics from type 1. Similarly, the study reveals that the oldest TMPs, the most experienced and skilled, see more patients. This supposes that traditional medicine requires time to retain patients who would be more confident towards the most experienced. This attitude is also observed in conventional medicine, where rationality would like the patient to be reassured by the doctor's experience before being consulted, especially for specific pathologies.

## 5. Conclusion

Several plants are used in Kinshasa to manage diabetes mellitus by herbalists as well as by people with diabetes and traditional medicine practitioners. This study highlights not only antidiabetic recipes but also plants reported only in Kinshasa for the management of diabetes. It also evokes their usual values in this environment. It opens the way for a subsequent study to validate the antidiabetic use, particularly of particular species of the environment and those of specific special interest there.

## Figures and Tables

**Table 1:** Botanical characteristics and previous knowledge of the plants inventoried.

Taxon	*F*_CR_ (*n* = 326)	Family	Local name	Ethnicity	A._U._	A_.D._
*Abelmoschus esculentus* (L.) Moench^X^	0.61	Malvaceae	Dongodongo	Lingala	Fr [11]	*In vivo* rat: 100–200 mg/kg [12]
*Acalypha paniculata* Miq.^W^	1.23	Euphorbiaceae	Kabobo	Bemba	Nr	Nr
Aframomum melegueta K.Schum^W^	2.15	Zingiberaceae	Mundongo	Lingala	Tb	*In vivo* rat: 150–400 mg/kg [13]
					Lv	*In vivo* rat: 200–400 mg/kg [14]
*Albizia adianthifolia* (Schumach.) W. Wight^W^	1.23	Fabaceae	Mulu	Kongo	Lv [15]	*In vivo* guinea pig: 500 mg/kg [16]
*Alchornea cordifolia* (Schumach. and Thonn.) Müll. Arg.^Y^	0.92	Euphorbiaceae	Mambunzila	Kongo	Lv [17]	*In vivo* rat: 200–600 mg/kg [18]
*Aloe succotrina* Weston^V^	1.53	Asphodelaceae	Langa	Ngwaka	Lv [19]	*In vivo* rat: 100–200 mg/kg [20]
*Anacardium occidentale* L.^X^	0.92	Anacardiaceae	Nkasu	Kongo	Ro [21]	*In vitro*: 12.5 *µ*g/mL [22]
					Lv [23]	*In vivo* rat: 100 mg/kg [24]
					Sb	*In vivo* rat: 200 mg/kg [25]
*Annona senegalensis* Pers.^W^	3.07	Annonaceae	Kilolo	Kongo	Lv [8]	*In vivo* rat: 100–200 mg/kg [26]
*Antidesma venosum* E. Mey. ex Tul.^Y^	1.53	Phyllanthaceae	Misengo	Kongo	Sb [15, 27]	Nr
*Artemisia afra* Jacq. ex Willd.^X^	1.53	Asteraceae	Lengana	Bemba	Ap [28]	*In vivo* rat: 500–1000 mg/kg [29]
*Asparagus racemosus* Willd.^Y^	1.23	Asparagaceae	Lutwa	Mbala	Wp [30]	*In vitro* amylase model [31]
*Azadirachta indica* A. Juss.^V^	0.61	Meliaceae	Marumaru	Shi	Sd [8]	*In vivo* rat: 500 mg/kg [32]
					Ro	*In vivo* rat:100–800 mg/kg [33]
*Baphia capparidifolia* Baker^X^	5.52	Fabaceae	Mumbwo	Ngbandi	Nr	Nr
*Bidens pilosa* L.^Z^	5.52	Asteraceae	Kokoyalimo	Lokele	Wp [15]	*In vivo* rat: 250 mg/kg [34]
*Boerhavia diffusa* L.^Y^	2.45	Nyctaginaceae	Ndemba	Ngwaka	L [35]	*In vivo* rat: 500 mg/kg [36]
					Ro	*In vivo* rat: 50–300 mg/kg [37]
*Brassica juncea* (L.) Czern.^W^	3.68	Brassicaceae	Nkofi	Kongo	Sd [38]	*In vivo* rat: 250–450 mg/kg [39]
						*In vivo* mice: 100 mg/kg [40]
					Lv	*In vitro* and *in vivo* rat: 500 mg/kg [41]
*Bridelia ferruginea* Benth.^Z^	3.37	Phyllanthaceae	Kimwindu kinseke	Kongo	Rb [42]	*In vivo* rat: 50 mg/kg [43]
					Lv	*In vivo* rat: 200–800 mg/kg [44]
*Brillantaisia owariensis* P. Beauv.^Y^	1.23	Acanthaceae	Lembalemba	Lingala	Nr [45]	*In vivo* rat: 500–2,000 mg/kg [46].
*Cajanus cajan* (L.) Huth^Z^	5.52	Fabaceae	Ngoliolio	Ngwaka	Lv [35]	*In vivo* rat: 400–600 mg/kg [47]
*Catharanthus roseus* (L.) G. Don^V^	1.23	Apocynaceae	Fulele	Ngwaka	Tb [48]	*In vivo* rat: 37.5–150 mg/kg [49]
*Chromolaena corymbosa* (Aubl.) R. M. King and H. Rob.^X^	6.44	Asteraceae	Mpala kasakula	Kongo	Nr	Nr
*Citrus* × *aurantium* L.^W^	1.84	Rutaceae	Nlala	Kongo	Fr [15]	*In vivo* rat: 200–300 mg/kg [50]
*Costus lucanusianus* J. Braun and K. Schum.^Y^	3.37	Costaceae	Ngo nkini	Kongo	Lv	*In vivo* rat: 100–200 mg/kg [51]
*Costus phyllocephalus* K. Schum.^Z^	1.23	Costaceae	Mafulungu	Kongo	Lv [52]	Nr
*Crinum ornatum* (Aiton) Herb.^Z^	1.84	Amaryllidaceae	Munselebende	Kongo	Nr [38]	Nr
*Crossopteryx febrifuga* (Afzel. ex G. Don) Benth.^W^	4.91	Rubiaceae	Mvala	Kongo	Sb [15]	*In vivo* rat: 500–1500 mg/kg [53]
*Crotalaria medicaginea* Lam.^Z^	1.84	Fabaceae	Nkeka zango	Yanzi	Nr	Nr
*Cymbopogon citratus* (D. C.) Stapf^W^	4.60	Poaceae	Sinda	Lingala	Wp [8]	*In vivo* rat: 2500–5000 mg/kg [54]
*Dioscorea dumetorum* (Kunth) Pax^X^	5.83	Dioscoreaceae	Ngamba	Kongo	Bb [55]	*In vivo* rat: 100 mg/kg [56]
*Diospyros heudelotii* Hiern^W^	3.68	Ebenaceae	Lufwa lundomba	Kongo	Ro [52]	Nr
*Dysphania ambrosioides* (L.)^x^	9.51	Amaranthaceae	Kulamoka	Yaka	Lv [7]	*In vivo* rat: 100–400 mg/kg [57]
*Erythrina abyssinica* Lam.^w^	1.23	Fabaceae	Kikumbu	Kongo	Rb [8]	*In vivo* guinea pig: 500 mg/kg [16]
*Ficus benghalensis* L.^Y^	1.53	Moraceae	Nsanda	Suku	Sb [8]	*In vitro*: AC _50_ = 84.44 ± 1.65 *μ*g/mL [58]
*Ficus exasperata* Vahl^Y^	1.23	Moraceae	Kikuya	Kongo	Lv [38]	*In vivo* rat: 150 mg/kg [59]
*Garcinia kola* Heckel^V^	18.40	Clusiaceae	Ngadiadia	Kongo	Sd [60]	*In vivo* rat: 200–800 mg/kg [61]
*Gladiolus gregarius* Welw. ex Baker^W^	0.61	Iridaceae	Litungulu ya Zamba	Lingala	Bb [15]	Nr
*Gymnanthemum amygdalinum* (Delile) Sch. Bip.^V^	3.07	Asteraceae	Mukari kari	Kongo	Lv [62]	*In vitro*-*α*-glucosidase: IC_50_ 47.29 ± 1.12 *µ*g/mL [63]
*Vernonia amygdalina* Delile (*Synonym*)						
*Harungana madagascariensis* Lam. ex Poir.^Y^	2.45	Hypericaceae	Ntumu	Mbala	Lv [15]	*In vivo* rat: 500 mg/kg [64]
*Heinsia crinita* (Wennberg) G. Taylor^Z^	2.76	Rubiaceae Juss.	Iyaku	Mongo	Lv [65]	*In vivo* rat: 400 mg/kg [66]
*Hymenocardia acida* Tul.^X^	4.29	Phyllanthaceae	Kigeti	Kongo	Lv [15]	F: *in vivo* rat: 250–1000 mg/kg [67]
*Lantana camara* L.^W^	0.61	Verbenaceae	Ngbanyamando	Ngwaka	Lv [15]	*In vivo* rat: 200–400 mg/kg [68]
					Fr	*In vivo* rat: 50–2000 mg/kg [69]
*Lippia multiflora* Moldenke^Y^	1.23	Verbenaceae	Bulukutu	Kongo	Lv [52]	Nr
*Marsdenia latifolia* (Benth.) K. Schum.^W^	0.92	Apocynaceae	Lolango	Kongo	Nr [70]	*In vivo* rat: [71]
*Gongronema latifolium* Benth. (Synonym)						
*Millettia drastica* Welw. ex Baker^W^	0.92	Fabaceae	Mwengzti	Kongo	Ro [52]	Nr
*Mitragyna stipulosa* (DC.) Kuntze^W^	1.23	Rubiaceae	Liluku	Lingala	Nr [72]	Nr
*Monodora myristica* (Gaertn.) Dunal^Y^	2.45	Annonaceae	Mpei	Kongo	Sd [73]	*In vivo* rat: 50–200 mg/kg [74]
*Morinda lucida* Benth.^Y^	1.23	Rubiaceae	Nkisi	Kongo	Lv [8]	*In vitro*-*α*-glucosidase: [75]
					Sb	*In vivo* rat: 125–500 mg/kg [76]
*Musanga cecropioides* R. Br. ex Tedlie^W^	1.23	Urticaceae	Nsanga	Kongo	Lv [52]	*In vivo* rat: 10–40 mg/kg [77]
					Sb	*In vitro α*-glucosidase [78]
*Nauclea latifolia* Sm.^Y^	1.53	Rubiaceae	Kilolo	Kongo	Lv [79]	*In vivo* rat: 100–200 mg/kg [80]
*Ocimum gratissimum* L.^Y^	1.23	Lamiaceae	Dinsusu-nsusu	Kongo	Lv [81]	*In vitro α*-glucosidase [78]
*Palisota schweinfurthii* C. B. Clarke^X^	8.28	Commelinaceae Mirb	Mabongubongu	Yanzi	Lv [52]	Nr
					Wp [52]	Nr
*Pentaclethra macrophylla* Benth.^X^	1.84	Fabaceae	Mutunzama	Kongo	Sb [45]	*In vivo* rat: 200–400 mg/kg [82]
*Persea americana* Mill.^X^	30.67	Lauraceae	Savoka	Yanzi	Lv [83]	*In vivo* rat: 100 mg/kg [84]
					Fr	*In vitro α*-glucosidase [85]
*Phaseolus vulgaris* L.^W^	1.53	Fabaceae	Madesu	Lingala	Sd [86]	*In vivo* rat: 1–5 mg/kg [87]
*Phyllanthus niruri* L.^Y^	2.45	Phyllanthaceae	Kahungahunga	Luba-kas	Ro [88]	*In vivo* rat: 200–400 mg/kg [89]
					Ap	*In vitro α*-glucosidase: [90]
*Piliostigma reticulatum* (DC.) Hochst.^X^	17.79	Fabaceae	Falabuta	Suku	Lv [91]	*In vivo* rat: 250–1000 mg/kg [92]
*Piliostigma thonningii* (Schumach.)^Y^	1.53	Fabaceae	Tshifumbe	Luba-kas	Sb [15]	*In vivo* rat: 500 mg/kg [93]
*Piper guineense* Schumach. and Thonn.^W^	0.61	Piperaceae	Kapinde	Kongo	Lv [52]	*In vivo* rat: 40–100 mg/kg [94]
*Platymitra arborea* (Blanco) P. J. A. Kessler^Y^	2.45	Annonaceae	Mpei	Kongo	Nr	Nr
*Psidium guajava* L.^W^	0.61	Myrtaceae	Mapela	Lingala	Lv [79]	*In vivo* rat: 40–100 mg/kg [94]
*Quassia amara* L.^X^	0.61	Simaroubaceae	Mupesipesi	Luba-kat	Lv, T, Ro [95]	*In vivo* rat: 200–500 mg/kg [96]
*Rauvolfia mannii* Stapf^X^	0.92	Apocynaceae	Kilungu	Kongo	Nr	Nr
*Schwenckia americana* L.^Y^	2.45	Solanaceae	Lunzilanzila	Kongo	Wp [45]	*In vivo* rat: 600 mg/kg [97]
*Senna alata* (L.) Roxb.^X^	30.67	Fabaceae	Mbwabwa	Kongo	Lv [98]	*In vivo* rat: 500–500 mg/kg [99]
					Fr [100]	*in vivo*: 250–500 mg/kg [101]
*Senna occidentalis* (L.) Link^V^	2.15	Fabaceae	Ntsambi ntsambi	Kongo	Lv [102]	*In vivo* rat: supplement at doses of 12.5%, 25%, and 50% [103]
					Ro	*In vivo* rat: 400 mg/kg [104]
*Syzygium cordatum* Hochst. ex Krauss^Y^	1.53	Myrtaceae	Mugorhe	Shi	Lv [105]	*In vivo* rat: 3–50 mg/kg [106]
*Tephrosia vogelii* Hook. f.^X^	7.98	Fabaceae	Uleku	Kongo	Nr [52]	Nr
*Terminalia mollis* M. A. Lawson^Y^	2.45	Combretaceae	Mboumbou	Luba-kas	Lv [15]	Nr
					Ro [15]	Nr
*Vachellia karroo* (Hayne) Banfi and Galasso^X^	17.79	Fabaceae	Munga	Luba-kat	Lv [15]	*In vitro α*-glucosidase: 25 *μ*g/mL [107]
Acacia karroo Hayne (*Synonym*)						
*Voacanga africana* Stapf^W^	0.61	Apocynaceae	Kondiankondia	Yaka	Lv [79]	*In silico* [108]
*Ziziphus mucronata* Willd.^W^	1.23	Rhamnaceae	Sula	Hemba	Lv	*In vitro α*-glucosidase: 1 mg/mL, A: 70% [109]
					Sb	*In vitro α*-glucosidase: 62.5 *µ*g/mL, A: 20% [110]
					Ro [111]	Rat: 150–300 mg/kg [112]

A_.U._: antidiabetic use, A_.D._: previous antidiabetic activity, *F*_RC_: relative frequency of citation in percentage, Bb: bulb, Rb: root bark, Sb: stem bark, Lv: leaves, Fr: fruits, Sd: seeds, and Ap: aerial parts.

**Table 2:** Antidiabetic recipes in monophytotherapy and other indications.

Plant species	*R*_EC_	UP	Preparation	*E*_*R*_	Eesp	ICR	ICp	IFR	UVO	UVP	Other indications
*Abelmoschus esculentus* ^X^	R1	Lv	Maceration	2	2	0.01	0.01	1.0	0.04	0.04	Constipation
*Acalypha paniculata* ^W^	R2	Rb	Decoction	4	4	0.01	0.01	1.0	0.04	0.04	Amoeba
*Aframomum melegueta* ^W^	R3	Sd	Infusion	7	7	0.02	0.02	1.0	0.04	0.04	Gastroesophageal reflux
*Albizia adianthifolia* ^W^	R4	Lv	Maceration	4	4	0.01	0.01	1.0	0.06	0.06	Sexual weakness and sores
*Alchornea cordifolia* ^Y^	R5	Ro	Decoction	3	3	0.01	0.01	1.0	0.06	0.06	Malaria, stomach aches
*Aloe succotrina* ^V^	R6	Lv	Infusion	5	5	0.02	0.02	1.0	0.08	0.08	Acne, malaria, and wounds
*Anacardium occidentale* ^X^	R7	Lv	Maceration	3	3	0.01	0.01	1.0	0.04	0.04	Warts
*Annona senegalensis* ^W^	R8	Sb	Decoction	8	10	0.02	0.03	0.8	0.10	0.12	Hepatitis, malaria, sexual impotence, and sores
	R9	Ro	Decoction	2		0.01		0.2	0.04		Urogenital infections
*Antidesma venosum* ^Y^	R10	Ro	Decoction	5	5	0.02	0.02	1.0	0.08	0.08	Chest pain, pneumonia, and cough
*Artemisia afra* ^X^	R11	Lv	Maceration	5	5	0.02	0.02	1.0	0.04	0.04	Malaria
*Asparagus racemosus* ^Y^	R12	Wp	Decoction	4	4	0.01	0.01	1.0	0.04	0.04	Joint stiffness
*Azadirachta indica* ^V^	R13	Lv	Maceration	2	2	0.01	0.01	1.0	0.04	0.04	Diarrhea, malaria, and stomachaches
*Baphia capparidifolia* ^X^	R14	Lv	Infusion	18	18	0.06	0.06	1.0	0.04	0.04	Inflammation
*Bidens pilosa* ^Z^	R15	Ro	Infusion	14	18	0.04	0.06	0.8	0.04	0.08	Cataract
	R16	Fr	Decoction	2		0.01		0.1	0.04		Glaucoma
	R17	Lv	Maceration	2		0.01		0.1	0.04		Pyomyositis
*Boerhavia diffusa* ^Y^	R18	Lv	Maceration	8	8	0.02	0.02	1.0	0.04	0.04	Angina
*Brassica juncea* ^W^	R19	Lv	Infusion	12	12	0.04	0.04	1.0	0.04	0.04	Eczema
*Bridelia ferruginea* ^Z^	R20	Rb	Decoction	11	11	0.03	0.03	1.0	0.04	0.04	Dislocation
*Brillantaisia owariensis* ^Y^	R21	Rb	Decoction	4	4	0.01	0.01	1.0	0.04	0.04	Epilepsy
*Cajanus cajan* ^Z^	R22	Lv	Maceration	18	18	0.06	0.06	1.0	0.04	0.04	Malaria
*Catharanthus roseus* ^V^	R23	Lv	Infusion	4	4	0.01	0.01	1.0	0.04	0.04	High blood pressure
*Chromolaena corymbosa* ^X^	R24	Lv	Maceration	21	21	0.06	0.06	1.0	0.04	0.04	Tuberculosis
*Citrus × aurantium* ^W^	R25	Lv	Infusion	6	6	0.02	0.02	1.0	0.04	0.04	Colic
*Costus lucanusianus* ^Z^	R26	Lv	Maceration	11	11	0.03	0.03	1.0	0.04	0.04	Peptic ulcer disease
*Costus phyllocephalus* ^Y^	R27	Lv	Decoction	4	4	0.01	0.01	1.0	0.04	0.04	Cough
*Crinum ornatum* ^Z^	R28	Lv	Maceration	6	6	0.02	0.02	1.0	0.04	0.04	Conjunctivitis
*Crossopteryx febrifuga* ^W^	R29	Ro	Decoction	14	16	0.04	0.05	0.9	0.06	0.06	Diarrhea and malaria
	R30	Lv	Decoction	2		0.01		0.1	0.06		Diarrhea and malaria
*Crotalaria medicaginea* ^Z^	R31	Wp	Infusion	4	4	0.01	0.01	1.0	0.04	0.04	Cough
*Cymbopogon citratus* ^W^	R32	Lv	Maceration	15	15	0.05	0.05	1.0	0.04	0.04	Malaria and cough
*Dioscorea dumetorum* ^X^	R33	Tb	Decoction	19	19	0.06	0.06	1.0	0.06	0.06	Diarrhea and wound
*Diospyros heudelotii* ^W^	R34	Ro	Decoction	12	12	0.04	0.04	1.0	0.04	0.04	Contusion
*Dysphania ambrosioides* ^X^	R35	Wp	Decoction	31	31	0.10	0.10	1.0	0.06	0.06	Intestinal bruising and worms
*Erythrina abyssinica* ^W^	R36	Rb	Decoction	2	4	0.01	0.01	0.5	0.06	0.12	Sexual weakness and malaria
	R37	Lv	Decoction	2		0.01		0.5	0.08		Cancer, cough, and tuberculosis
*Ficus benghalensis* ^Y^	R38	Sb	Decoction	5	5	0.02	0.02	1.0	0.06	0.06	Eczema and vomiting
*Ficus exasperata* ^Y^	R39	Lv	Infusion	4	4	0.01	0.01	1.0	0.06	0.06	Convulsions and peptic ulcer disease
*Garcinia kola* ^V^	R40	Sd	Infusion	60	60	0.18	0.18	1.0	0.04	0.04	Glaucoma
*Gladiolus gregarius* ^W^	R41	Bb	Infusion	2	2	0.01	0.01	1.0	0.06	0.06	Angina and amoeba
*Gymnanthemum amygdalinum* ^V^	R42	Lv	Decoction	10	10	0.03	0.03	1.0	0.10	0.10	High blood pressure, STI, malaria, and cough
*Harungana madagascariensis* ^Y^	R43	Rb	Decoction	8	8	0.02	0.02	1.0	0.06	0.06	Sickle cell disease and malaria
*Heinsia crinita* ^Z^	R44	Lv	Decoction	9	9	0.03	0.03	1.0	0.06	0.06	Cough and HIV
*Hymenocardia acida* ^X^	R45	Ro	Decoction	14	14	0.04	0.04	1.0	0.04	0.04	Sickle cell disease
*Lantana camara* ^W^	R46	Fr	Decoction	12	12	0.04	0.04	1.0	0.06	0.06	Malaria and cough
*Lippia multiflora* ^Y^	R47	Lv	Maceration	4	4	0.01	0.01	1.0	0.08	0.08	Angina, asthma, and sexual weakness
*Marsdenia latifolia* ^W^	R48	Ro	Decoction	3	3	0.01	0.01	1.0	0.08	0.08	Constipation, cough, and intestinal worms
*Millettia drastica* ^W^	R49	Ro	Decoction	3	3	0.01	0.01	1.0	0.04	0.04	Sickle cell disease
*Mitragyna stipulosa* ^W^	R50	Rb	Decoction	4	4	0.01	0.01	1.0	0.04	0.04	Urogenital infections
	R51	Sd	Decoction	5	8	0.02	0.02	0.6	0.04	0.08	High blood pressure
	R52	Lv	Maceration	2		0.01		0.3	0.04		Anthrax
	R53	Fr	Infusion	1		0.00		0.1	0.04		Cancer
*Morinda lucida* ^Y^	R54	Lv	Maceration	4	4	0.01	0.01	1.0	0; 04	0.04	Cancer
*Musanga cecropioides* ^W^	R55	Lv	Maceration	4	4	0.01	0.01	1.0	0.08	0.08	High blood Pressure, cough, and tuberculosis
*Nauclea latifolia* ^Y^	R56	Lv	Decoction	3	5	0.01	0.02	0.6	0.08	0.12	Malaria, toothache, and sore
	R57	Ro	Decoction	2		0.01		0.4	0.06		Bronchitis and dysentery
*Ocimum gratissimum* ^Y^	R58	Lv	Decoction	4	4	0.01	0.01	1.0	0.08	0.08	Diarrhea, wound, and sinusitis
*Palisota schweinfurthii* ^X^	R59	Lv	Maceration	27	27	0.08	0.08	1.0	0.06	0.06	Snakebite and rheumatism
*Pentaclethra macrophylla* ^W^	R60	Rb	Decoction	6	6	0.02	0.02	1.0	0.04	0.04	Amoeba
*Persea americana* ^X^	R61	Lv	Maceration	100	100	0.31	0.31	1.0	0.04	0.04	High blood pressure
*Phaseolus vulgaris* ^W^	R62	Sd	Infusion	5	5	0.02	0.02	1.0	0.06	0.06	Wounds and ulcer
*Phyllanthus niruri* ^Y^	R63	Sb	Maceration	6	8	0.02	0.02	0.8	0.04	0.08	Wounds
	R64	Ap	Infusion	2		0.01		0.3	0.06		Urogenital infections and ulcers
*Piliostigma reticulatum* ^X^	R65	Rb	Decoction	58	58	0.18	0.18	1.0	0.08	0.08	Bronchitis, headaches, and rheumatism
*Piliostigma thonningii* ^Y^	R66	Rb	Decoction	5	5	0.02	0.02	1.0	0.08	0.08	Diarrhea, wound, and ulcer
*Piper guineense* ^W^	R67	Fr	Infusion	2	2	0.01	0.01	1.0	0.04	0.04	Asthma
*Platymitra arborea* ^Y^	R68	Fr	Maceration	8	8	0.02	0.02	1.0	0.02	0.02	NR
*Psidium guajava* ^W^	R69	Lv	Maceration	2	2	0.01	0.01	1.0	0.08	0.08	Diarrhea, hepatitis, and malaria
*Quassia amara* ^X^	R74	Ro	Decoction	2	2	0.01	0.01	1.0	0.10	0.10	Malaria, sexual weakness, typhoid, and rheumatism
*Rauvolfia mannii* ^X^	R70	Lv	Maceration	3	3	0.01	0.01	1.0	0.06	0.06	Jaundice and female infertility
*Schwenckia americana* ^Y^	R71	Wp	Decoction	8	8	0.02	0.02	1.0	0.06	0.06	Diarrhea and cholera
*Senna alata* ^X^	R72	Lv	Maceration	100	100	0.31	0.31	1.0	0.04	0.04	Constipation
*Senna occidentalis* ^V^	R73	Lv	Infusion	7	7	0.02	0.02	1.0	0.06	0.06	Sexual weakness and malaria
*Syzygium cordatum* ^*Y*^	R75	Lv	Maceration	5	5	0.02	0.02	1.0	0.06	0.06	Diarrhea and cough
*Tephrosia vogelii* ^X^	R76	Lv	Infusion	26	26	0.08	0.08	1.0	0.02	0.02	NR
*Terminalia mollis* ^Y^	R77	Lv	Maceration	8	8	0.02	0.02	1.0	0.08	0.08	Cough ulcers, measles, and gastroduodenal diseases
*Vachellia karroo* ^*X*^	R78	Ro	Decoction	58	58	0.18	0.18	1.0	0.08	0.08	Constipation, diarrhea, and cough
*Voacanga africana* ^W^	R79	Lv	Maceration	2	2	0.01	0.01	1.0	0.06	0.06	Sexual weakness and high blood pressure
*Ziziphus mucronata* ^W^	R80	Rb	Decoction	4	4	0.01	0.01	1.0	0.06	0.06	Angina and sickle cell disease

ER (number of citations per recipe); Eesp (number of citations per plant); CIR (consensus index of the recipe); CIp (consensus index of the plant); RRI (recipe reliability index); UVo (the usual value of the organ); UVp (the number of uses of the plant out of the number of uses of the set of species); NR (Nothing to report); Nu(number of uses); N (number of people consulted); Nu = 52; *N* = 326.

**Table 3:** Antidiabetic recipes that use two herbs in combination.

N°	Taxon 1	Taxon 2	No recipe	PU-ratio	Preparation	D.T. (D)	*F*_CR_
1	*Chromolaena corymbosa* ^X^	*Dioscorea dumetorum* ^X^	R81	F-F (1 ÷ 2)	Decoction	30	3.1
2	*Senna alata* ^X^	*Alchornea cordifolia* ^Y^	R82	F-R (1 ÷ 1)	Maceration	45	3.7
3	*Morinda lucida* ^Y^	*Nauclea latifolia* ^Y^	R83	F-R (2 ÷ 1)	Infusion	30	2.5
4	*Platymitra arborea* ^Y^	*Schwenckia americana* ^Y^	R84	R-R (1 ÷ 1)	Decoction	45	3.7
5	*Albizia adianthifolia* ^W^	*Annona senegalensis* ^W^	R85	F-R (2 ÷ 1)	Decoction	45	4.6
6	*Garcinia kola* ^V^	*Gymnanthemum amygdalinum* ^*V*^	R86	F-F (2 ÷ 1)	Decoction	45	1.8

PU-ratio: part used and proportion of the mixture; F: leaf; R: root; D.T.: duration of treatment; D: day; the F_CR_ is expressed in % (*n* = 326); N° recipe = antidiabetic recipe.

**Table 4:** General characteristics of the subjects consulted.

Class	Category	People with diabetes (*n* = 126)		Herbalists (*n* = 80)		TMP (*n* = 120)		Total % (*n* = 326)
		*E*	%	*E*	%	*E*	%	
Age	18–28	3	2.4	6	8	7	5.8	4.9
	28–38	21	16.7	6	8	18	15.0	13.8
	38–48	7	5.6	12	15	27	22.5	14.1
	48–58	35	27.8	41	51	60	50.0	41.7
	>58	60	47.6	15	19	8	6.7	25.4

Experience (year)	1–5	10	7.9	14	18	10	8.3	12.9
	6–10	15	11.9	7	9	58	48.3	24.5
	11–15	20	15.9	40	50	26	21.7	26.4
	16–20	78	61.9	8	10	20	16.7	32.5
	21–25	3	2.4	11	14	6	5.0	6.1

Profession	Trade	21	16.7	68	85	4	3.3	28.5
	Public sector	3	2.4	0	0	5	4.2	2.5
	Liberal	57	45.2	12	15	10	8.3	24.2
	Housework	45	35.7	0	0	3	2.5	14.7
	TMP	0	0.0	0	0	98	81.7	30.1

Gender	Female	84	66.7	64	80	55	45.8	62.3
	Male	42	33.3	16	20	65	54.2	37.7

Habitation	Bumbu	15	11.9	16	20	32	26.7	19.3
	Kalamu	45	35.7	23	29	29	24.2	29.8
	Lemba	11	8.7	10	13	6	5.0	8.3
	Limete	25	19.8	10	13	8	6.7	13.2
	Makala	30	23.8	21	26	45	37.5	29.4

Level of studies	None	2	1.6	20	25	6	5.0	8.6
	Primary	21	16.7	15	19	18	15.0	16.6
	Professional	7	5.6	3	4	10	8.3	6.1
	Secondary	47	37.3	41	51	66	55.0	47.2
	University	49	38.9	1	1	20	16.7	21.5

TMP: practitioner of traditional medicine; experience in the diabetic category is the number of years since the patient was diagnosed with diabetes.

## Data Availability

The data used in this study are included within the article.
